# Comparison of the biomechanical characteristics of human ovarian tissue after vitrification *versus* slow freezing

**DOI:** 10.5935/1518-0557.20250045

**Published:** 2025

**Authors:** Marta Méndez, Carolina Herranz-Diez, Santiago González, Janisse Ferreri, Josep Maria Calafell, Jordi Otero, Ramon Farre, Salvadora Civico, Francesc Fabregues

**Affiliations:** 1 Institute of Gynaecology Obstetrics and Neonatology of Hospital Clínic de Barcelona Spain; 2 Biophysics and Bioengineering Unit, Faculty of Medicine and Health Sciences, University of Barcelona, Barcelona, Spain; 3 BCNatal, Sant Joan de Déu University Hospital, Barcelona, Spain; 4 CEFER Reproduction Institute, Barcelona, Spain

**Keywords:** ovarian cryopreservation, vitrification, slow freezing, mechanobiology, tissue stiffness

## Abstract

**Objective:**

Numerous studies have compared ovarian tissue cryopreservation methods, including slow freezing/rapid thawing (SF/RT) and vitrification/warming (V/W), focusing on morpho-functional status, follicle density, stromal cell integrity, and gene expression during in vitro culture. However, results remain inconclusive. This study aims to evaluate the effects of SF/RT and V/W on the ovarian cortex’s biomechanical properties.

**Methods:**

Human ovarian biopsies were taken from five women between 32 and 45 years undergoing laparoscopic surgery for tubal sterilization. For each patient, one small sample of fresh tissue was used as control, and the remaining tissue were vitrified or frozen by slow freezing method. The tissue stiffness of the cryopreserved samples at the micrometer scale was measured by Atomic Force Microscopy after thawing and warming, depending of the cryopreservation method.

**Results:**

The median stiffness of the ovarian cortex was 3670.00 Pa (Pascal) (IQR 2146.4) in the control group. After cryopreservation, the median stiffness slightly decreased to 1305.90 Pa (IQR 503.51) with SF/RT and to 2284.50 Pa (IQR 3314.40) with V/W. General linear model analysis revealed no significant effect of cryopreservation method on the ovarian cortex stiffness (F=2.750, p=0.071). No significant differences were observed based on the intra-sample zone studied by AFM. However, a significant inter-patient effect on tissue stiffness was identified (F=3.958, *p*=0.006).

**Conclusions:**

The study findings suggest that ovarian tissue freezing methods do not have a relevant impact on functional aspects of the extracellular matrix (ECM), suggesting that given the logistical advantages of vitrification, this technique should be prioritized.

## INTRODUCTION

Ovarian tissue cryopreservation (OTC) is an accepted technique as a method of fertility-preservation and is currently no longer considered experimental (Practice Committee of the American Society for Reproductive Medicine, 2019). This approach would be advised for prepubertal patients and individuals for whom the deferral of cancer treatment is impractical, precluding the option of undergoing conventional ovarian stimulation and oocyte retrieval procedures (Guideline Group on Female Fertility Preservation *et al.*, 2020).

A substantial expertise in the fields of ovarian tissue cryopreservation (OTC) and subsequent transplantation (OTT) have been demonstrated in recent studies. Notably, they have evidenced a reactivation of ovarian function within a span of 4-5 months, with pregnancy rates of 37% and live birth rates of 28%. Furthermore, the average period of functionality for the transplanted tissue has been suggested to extend between 2.5 and 5 years (Dolmans *et al.*, 2021; Khattak *et al.*, 2022). The number of primordial follicles that are present in ovarian fragments at the time of transplantation and that survive the grafting procedure appears to be the major determinant of OTT success and graft longevity (Roness & Meirow, 2019; Silber *et al.*, 2018). Therefore, age and ovarian reserve markers will be determining factors in the effectiveness of the technique (Guideline Group on Female Fertility Preservation *et al.*, 2020; Practice Committee of the American Society for Reproductive Medicine, 2019). This would be related to the fact that there is a loss of primordial follicles between 50-90% after the transplant (Dolmans *et al.*, 2007; Gavish *et al.*, 2018). This loss has been linked to the effect of cryopreservation (Gook *et al.*, 1999) and also to the revascularization of the tissue once transplanted (Liu *et al.*, 2002). The two common methods for ovarian tissue cryopreservation are slow freezing/rapid thawing (SF/RT) and vitrification/warming (V/W) procedures. Both methods have been shown to result in more cytotoxicity and less follicular growth when compared to fresh controls (Oktem *et al.*, 2011).

Nevertheless, there is more experience with the use of the slow freezing method and in fact, most of the achieved pregnancies come from the rapid thawing protocol. However, recent data show a similar pregnancy rate after the vitrification/thawing protocol (Khattak *et al.*, 2022).

Several studies have attempted to compare both methods of ovarian tissue cryopreservation, focusing, on the one hand, on the post SF/RT and V/W pre-graft analysis, studying the morpho-functional status of tissue, the density of primordial follicles, the proportion of intact follicles, the proportion of intact stromal cells, and gene expression during in vitro culture (Behl *et al.*, 2023; Fabbri *et al.*, 2016; Keros *et al.*, 2009; Wang *et al.*, 2016). On the other hand, both techniques have also been compared after xenotransplantation, analyzing follicular survival parameters (Amorim *et al.*, 2012; Lee *et al.*, 2019; Rahimi *et al.*, 2009). Altogether, the results are still controversial.

A novel aspect in the area within ovarian physiology is mechanobiology, which attempts to elucidate the indispensable role of tissue biomechanical properties for its correct functioning and how modifications to these characteristics can give rise to pathological states (Bissell *et al.*, 1982; Ingber, 2006). Despite the comprehensive investigation into the hormonal regulation of folliculogenesis, the involvement of mechanical signaling in this process has not been as thoroughly examined. Increasing evidence suggests that the three-dimensional structure and mechanical characteristics of the extracellular matrix (ECM) in the ovary, including factors like its mechanical stiffness, significantly influence follicle development. These properties exert a direct impact on cellular processes such as proliferation, differentiation, as well as the production and diffusion of growth factors (Hornick *et al.*, 2012; Jorge *et al.*, 2014). In this line, over the last decade there has been an escalating interest in unraveling the mechanobiological intricacies of the human ovary. This entails a comprehensive examination of the dynamic interplay existing between ovarian cells and their encompassing microenvironment (Thorne *et al.*, 2015; Woodruff & Shea, 2011).

Concretely, recent data have shown changes in components of the ovarian ECM throughout a woman’s life and have suggested that the biomechanical properties of the ovarian cortex are crucial in ovarian aging (Grosbois *et al.*, 2023; Ouni *et al.*, 2020, 2021).

The inherent dependence of tissue elasticity on its ECM underscores the necessity for a direct characterization method. This is most effectively accomplished through the assessment of Young’s Modulus (E), a parameter defining the sample’s stiffness or resistance to deformation (Johnson, 1985). Noteworthy, Atomic Force Microscopy (AFM) has emerged as a very suitable technique to assess E, by quantitatively probing the mechanical properties and forces at the nanometer or micrometer scales at which cells sense their mechanical microenvironment (Allison *et al.*, 2010; Jorba *et al.*, 2017).

Taking all of the above into account, the aim of this study was to compare the biomechanical characteristics of the ovarian cortex by AFM after SF/RT and V/W and also to analyze how both procedures have an impact on the biomechanics of the samples analyzed fresh (immediately after biopsy, without the use of cryoprotectants).

## MATERIAL AND METHODS

### Tissue donors

Ovarian tissue was collected as a small biopsy sample from 5 donors with a mean age of 38.5±5.1 (SD) years (median age 40.5, range 32 - 45 years). The biopsies were performed during laparoscopic surgery for tubal sterilization. Use of human ovarian tissue for this study was approved by the Ethics Research Committee from our Center (HCB/2017/0856). All women were informed about the ongoing project and they signed an informed consent form.

### Tissue preparation

**Figure 1:**
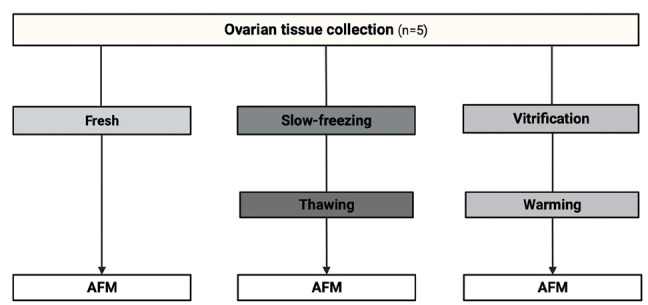
Experimental setup for comparing Young´s Modulus (E) by Atomic Force Microscopy (AFM) of the different cryopreservation protocols.

Excised ovarian tissue was placed in sterile 50 ml Falcon tubes (Becton Dickinson, Bedford MA, USA) containing 25 ml of buffer media (DPBS, Thermo Fisher Scientific) and immediately transported at room temperature to the laboratory. Then, the ovarian cortical tissue was manually dissected from medullar tissue and cut into fragments of about 3-5mm by scalpel. For each patient, one small fragment of fresh tissue was used as control for AFM analysis, and the remaining fragments of tissue were vitrified or frozen by slow freezing method. Th*e* ovarian cortical samples from each patient were processed at the same time for both cryopreservation procedures, stored in liquid nitrogen for 2-25 weeks, and warmed/thawed in parallel (Figure 1). Furthermore, the AFM experiments were also performed at the same time to homogenize experimental conditions.

### Slow freezing technique/ thawing procedure

The SF protocol was executed in accordance with a previously described methodology, with certain modifications as outlined elsewhere (Andersen *et al.*, 2008). The ovarian tissue fragments were placed in plastic cryovials (Nunc, Denmark) containing 1.8ml of freezing solution, consisting of 1.5M ethylene glycol (EG) (Merck, Darmstadt, Germany), 0.1M sucrose (Merck, Darmstadt, Germany) and 10 mg/ml of human serum albumin. The cryovials were subjected to continuous rotation within an ice bath, maintaining a temperature of 4°C, for a duration of 30 minutes. Subsequently, the specimens were introduced into a computerized programmable freezer (Planner K10, Planner Ltd., UK). The cooling regimen initiated from 0 to -9°C at a pace of 2°C per minute, at which point the initiation of ice nucleation (seeding) was manually prompted. Following a 10-minute holding period, the temperature was gradually decreased to -40°C at a rate of 0.3°C per minute, succeeded by a rapid descent from -40 to -140°C at a rate of -10°C per minute. Subsequent to a 10-minute period of temperature stabilization, the cryovials were placed into liquid nitrogen for storage.

For the rapid thawing procedure, the cryovials were immersed in a 37°C water bath for 5 min. The cryoprotectants were removed at room temperature by stepwise dilution of EG in the thawing solutions. The thawed vials were emptied into a solution comprising 0.75M ethylene glycol (EG), 0.25M sucrose, and 20% serum substitute. The sample underwent equilibration for 10 minutes in this solution, followed by an additional 10-minute equilibration in a solution containing 0.25M sucrose and 20% serum substitute. The final step involved dilution in buffer media, with a duration of 10 minutes at room temperature.

### Vitrification/warming procedure

The VT protocol of ovarian tissue pieces was performed according to the protocol described by Kagawa and coworkers with some adjustments (Kagawa *et al.*, 2009). Cortical fragments underwent incubation in an equilibration solution containing 7.5% ethylene glycol (Merck, Darmstadt), 7.5% dimethyl sulfoxide (DMSO, Merck, Darmstadt, Germany), and 20% human serum albumin (Grifols, Spain). This incubation occurred over a period of 25 minutes at room temperature. Subsequently, there was a 15-minute incubation in a vitrification solution comprising 20% ethylene glycol, 20% DMSO, and 0.5M sucrose. The samples were transferred to precooled 1.8 ml cryogenic vials promptly immersed directly into liquid nitrogen. For the warming procedure, the samples were immersed directly into 4ml of pre-warmed 37°C thawing solution (TS, Kitazato, Japan) for 1 min. Later, they were transferred into 4 ml of diluent solution DS (DS, Kitazato, Japan) for 5 min at room temperature, and washed twice in washing solution (WS,Kitazato, Japan) solution for 5 min and 5 min respectively. Finally, the tissue pieces were washed in buffer media.

### Extracellular matrix (ECM) micromechanics measurement by atomic force microscopy (AFM)

**Figure 2:**
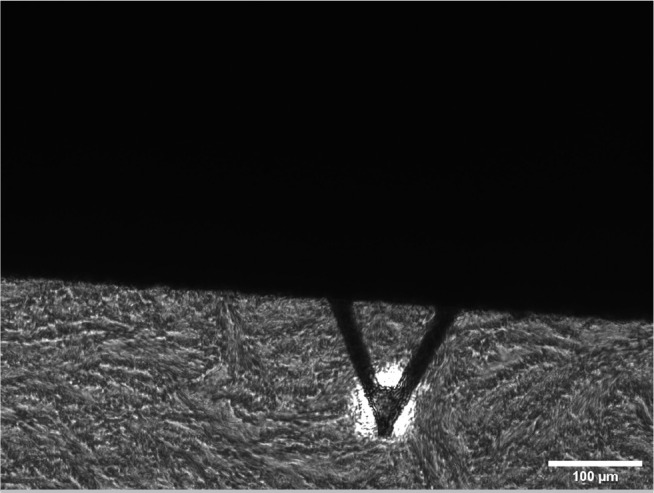
Transmission microscopic image of the AFM cantilever positioned on top ovarian tissue sample. Acronyms: AFM (Atomic Force Microscopy).

To perform the AFM measurements, an ovarian fragment of approximately 3-5 mm × 3-5 mm was placed on cold (4°C) saline physiological solution right after biopsy, after thawing or warming. Afterward, the ovarian sample was immersed in optimal cutting temperature compound (OCT, Sigma-Aldrich, Darmstadt, Germany) and quickly cryopreserved at -80 °C using a well-established protocol known for preserving the mechanical properties of ECM (Nonaka *et al.*, 2014). Thin slices of about 20 μm in thickness were obtained by cryosectioning (CM3050S, Leica Biosystems, Germany) and placed on top of positively charged glass slides. AFM measurements were conducted in PBS buffer at 37 °C, pH 7.4 using a custom built AFM mounted on an inverted optical microscope (TE2000,Nikon, Tokio, Japan). Force-displacement curves were acquired by indenting the tissue surface with a V-shaped silicon nitride cantilever (0.1 N/m of nominal spring constant) ended with a 2.25-μm radius spherical polystyrene bead (Novascan Technologies, Ames, IA) (Figure 2). The positioning of the cantilever was controlled by a piezoelectric actuator and evaluated through the use of strain gauge sensors (Physik Instrumente, Karlsruhe, Germany). Furthermore, a four-quadrant photodiode (S4349, Hamamatsu, Japan) was employed to quantify cantilever deflection, thereby determining the force exerted on the surface. The micromechanics of each tissue sample were probed in 5 randomly selected zones outside the follicle space (identified by microscope by its perifollicular fiber distribution) distributed among the whole tissue sample separated at least 0.2 mm from the edges. Twenty-five force curves (10 μm amplitude at a speed of 5 μm/s) were recorded for each zone in five different points randomly selected and separated at least 10 μm from each other. The elastic modulus was computed from the acquired force-displacement curves by adjusting the Hertz model at 1 μm of surface indentation as described by Otero and collaborators (Otero *et al.*, 2020) and expressed in Pascals (Pa).

### Statistical analysis

Continuous data are expressed as median with interquartile range (IQR) and categorical data as percentages if not stated differently.

A generalized linear model (GLM) was employed to evaluate the effect of the ovarian cryopreservation technique on ovarian stiffness, measured by Young’s elastic modulus. In this analysis, ovarian stiffness served as the dependent variable. Patient number identification and the number of replicate zones in the AFM measurements were incorporated as random effects within the model to evaluate intra-sample and inter-patient impact. The method of cryopreservation was included as a fixed effect. Breusch-Pagan test was used to assess heteroscedasticity.

*p-*value <0.05 was considered statistically significant. All analyses were performed using SPSS version 22.0.

## RESULTS

The mean age of patients at the time of the intervention was 38.5±5.1 (SD) years. Besides, the mean age at menarche was 12.0±1.0 (SD) years and the BMI of 30.7±3.1 (SD) kg/m^2^. The mean parity of the studied group was 2.0±1.3 (SD) child. One out of five women stated to be habitual smoker (Table 1).

**Table 1:** Clinical characteristics and origin of ovarian tissue of patients included in the study. BMI: body mass index.

	Age (years)	Smoker (1=yes; 0=no)	Age at menarche (years)	BMI (Kg/m^2^)	Parity (n)	Surgical procedure
**Patient 1**	45	0	12	35	4	Laparoscopy (Tubal sterilization)
**Patient 2**	41	0	11	30	1	Laparoscopy (Tubal sterilization)
**Patient 3**	32	0	14	32	3	Laparoscopy (Tubal sterilization)
**Patient 4**	40	0	11	27	1	Laparoscopy (Tubal sterilization)
**Patient 5**	35	1	12	28	2	Laparoscopy (Tubal sterilization)

**Figure 3:**
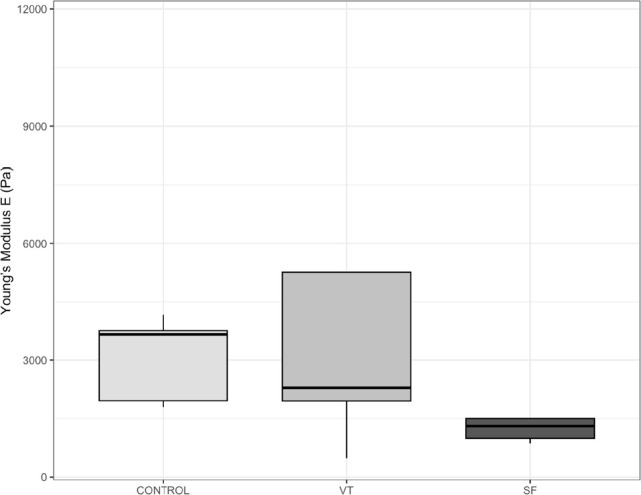
Young’s Modulus of ovarian cortex by AFM from the three experimental conditions: immediately after biopsy (Control), vitrification (VT) and slow-Freezing (SF).

Ovarian cortex stiffness results for each sample and experimental condition are presented in Table 2. In the control group, the highest ovarian cortex stiffness observed was 4170.00 Pa, while the lowest ovarian cortex stiffness observed was 1794.00 Pa. The median stiffness of the ovarian cortex, measured by AFM, was 3670.00 Pa (IQR 2146.4) in the control group. After cryopreservation with SF-RT, the median stiffness was 1305.90 Pa (IQR 503.51), and following VT-W, it was 2284.50 Pa (IQR 3314.40)(Figure 3). Following the GLM study, no significant effect was detected on the stiffness of the human ovarian cortex after cryopreservation by either slow freezing or vitrification (F=2.750, *p*-value 0.071), nor was any effect observed on tissue stiffness based on the intra-sample zone studied by AFM. However, a significant inter-patient effect on tissue stiffness was detected (F=3.958, *p*-value 0.006).

**Table 2:** Summary of the Young’s elastic Modulus (E) values obtained for each patient and condition by AFM in the ovarian cortex. Results are expressed as median (standard error).

	E Control (Pa)	E postwarming (Vitrification) (Pa)	E postthawing (Slow freezing) (Pa)
**Patient 1**	1954.1 (779.99)	1948.30 (458.58)	2384.9 (453.530)
**Patient 2**	1794.00 (375.43)	480.47 (137.22)	994.59 (653.19)
**Patient 3**	4170.00 (585.85)	2284.5 (602.71)	1498.1 (725.10)
**Patient 4**	4100.50 (2422.87)	9880.8 (2731.38)	1002.45 (808,38)
**Patient 5**	3670 (728.31)	5262.7 (1104.60)	1305.9 (1303.72)

## DISCUSSION

To the best of our knowledge, this is the first study that has proposed to analyze the biomechanical characteristics of the ovarian cortex by AFM after SF/RT and V/W procedures.

The results of this investigation suggest that both methods of ovarian tissue cryopreservation, vitrification and slow freezing, do not have a negative impact on the biomechanical characteristics of the cortex when measured by AFM and therefore, in principle, the components of the ECM responsible for rigidity and elasticity would not be affected by the freezing process. Accumulating evidence indicates the involvement of ovarian ECM in the intricate process of folliculogenesis, with a specific emphasis on primordial follicle activation.

The ECM elements, its structuring and also its distribution hold noteworthy significance in the context of folliculogenesis (Grosbois *et al.*, 2023; Ouni *et al.*, 2020, 2021).

The predominant theory suggests that the robust, collagen-enriched cortical region establishes a rigid physical context conducive to quiescence. Conversely, the softer medulla layer is thought to provide a more flexible environment, thereby facilitating the expansion and growth of follicles (Woodruff & Shea, 2011).

According to all this, it is plausible to hypothesize that the different processes that ovarian tissue undergoes during SF/RT and V/W could imply an effect on the components of the ECM and that they can be assessed by AFM.

Five patients of reproductive age were included in the study since previous studies that have studied the viscoelasticity of the ovarian cortex demonstrated that in prepubertal age and in established menopause the stiffness measured by AFM was significantly higher than that observed in reproductive age (Ouni *et al.*, 2021) . Although the age of the patients was higher than those who usually undergo cryopreservation of ovarian tissue, they were fertile patients and their inclusion in the study was appropriate. Furthermore, none of the patients were undergoing any hormonal treatment or were pregnant (if ovarian material had been obtained during cesarean section), since it has been shown that both situations could modify the biomechanical characteristics of the ovarian cortex. Nonetheless, the GLM analysis only identified an interpatient effect on ovarian tissue stiffness not the cryopreservation technique. This could explain the initial observed stiffness variation of the control samples, also been documented in other studies (Ouni *et al.*, 2021).

Experimental studies (Campos *et al.*, 2016) in human ovarian tissue have shown a loss of primordial follicles after different freezing and thawing processes (Oktem *et al.*, 2011). In addition, the clinical results show differences in favor of fresh transplantation (FT) *vs*. OTC with live birth rate 45% (95%. CI:23-86%) *vs*. 28%. (95%. CI: 24-34%) (Khattak *et al.*, 2022). However, in clinical practice OTC is required in most cases and therefore any analysis should focus on this scenario.

To date, three meta-analyses have attempted to compare the results of SF/RT vs. V/W freezing (Behl *et al.*, 2023; Shi *et al.*, 2017; Zhou *et al.*, 2016). The most recent one that includes a greater number of studies and analyzes more parameters (proportion of intact primordial follicles, proportion of intact stromal cells, proportion of DNA fragmentation in primordial follicles, and mean primordial follicle density), concludes that both cryopreservation methods affect the histological characteristics of the tissue compared to fresh samples. Nevertheless, no significant differences upon pooled analyses were observed between the two cryopreservation methods regarding all others parameters (Behl *et al.*, 2023). It is noteworthy that distinctions were exclusively reported in the proportion of undamaged stromal cells.

Four of the 19 studies included in the meta-analysis demonstrated a significantly greater proportion of intact stromal cells in V/W *versus* SF/RT (Chang *et al.*, 2011; Keros *et al.*, 2009; Wang *et al.*, 2008; Xiao *et al.*, 2010).

Some discordant data between the studies are related to: i) aspects related to the tissue source (ovaries of pregnant patients , transgender, pathologies, etc.) (Borrás *et al.*, 2022; Keros *et al.*, 2009; Sanfilippo *et al.*, 2015); ii) freezing methodological aspects (Amorim *et al.*, 2011; Rodriguez-Wallberg *et al*., 2012) and iii) data from tissue thawed and grafted into mice (Amorim *et al.*, 2012; Rahimi *et al.*, 2009). This condition could influence the results because it has recently been shown that vitrified-thawed human ovarian cortical tissue grafted to nude mice presented a marked increase in fibrosis, along with a decrease in cortical stroma content with a final alteration of follicular development (Kitajima *et al.*, 2022).

In this line, studies have indicated that the stroma of the ovarian cortex is more sensitive to damage caused by cryopreservation than the primordial follicles (Kim *et al.*, 2004). Therefore, the integrity of stromal cells has emerged as a key primary outcome.

The ovarian stroma contains cellular components of the immune system, ECM, fibroblasts and blood vessels and has been shown to promote growth of immature follicles (Biswas *et al.*, 2022).

Interstitial and perifollicular ECM components in the ovarian cortex have been examined, revealing variations depending on the follicular stage, the age of the patient and also hormonal therapies (Ouni *et al.*, 2020). These changes can modify the biomechanical characteristics (elasticity and viscoelasticity) that can be measured by AFM (Méndez *et al.*, 2022; Ouni *et al.*, 2021). Therefore, it is expected to find differences in AFM measurements inherent to each tissue sample. These differences are much smaller than the differences observed in different clinical conditions. The ECM is responsible for the viscoelasticity of the ovarian stroma and structural changes in it can directly affect folliculogenesis. Alterations in the ECM translate into an increase in stiffness, which is precisely what AFM analyzes.

The fact that the proportion of intact stromal cells is higher after vitrification than with slow freezing does not provide complete information about the ovarian stroma, since the ECM and its components directly influence the function of the stromal cells. The results of our study demonstrate that the functions of the ECM are not affected by both freezing techniques, therefore, they provide valuable information about this compartment of the ovarian cortex whose relationship with folliculogenesis is transcendental.

Unfortunately, the AFM analysis of the biomechanical characteristics of the ovarian cortex of the patients prevented histological studies from being carried out for conventional assessments. Unlike the data from Behl’s et al. meta-analysis in which the percentage of intact stromal cells was significantly higher after V/W (Behl *et al.*, 2023), our study demonstrates that overall there are no significant differences in the impact of both freezing methods on the biomechanical characteristics of the ovarian cortex measured by AFM.

Different methods have been used to assess the mechanical properties of tissues. Tensile (uniaxial/biaxial) or inflation testing have been extensively used to characterize the mechanical properties of advanced atherosclerotic plaques in porcine model (Chai *et al.*, 2014; Walsh *et al.*, 2014), however recent studies confirm that material testing by indentation such as AFM allows obtaining more detailed information on tissue stiffness and its correlation with the collagen of the plaques (Savvopoulos *et al.*, 2024). On the other hand, AFM has been used in animal models such as cats or cows to study the biomechanical characteristics of the ovarian cortex with satisfactory results (Pascoletti *et al.*, 2020; Stewart *et al.*, 2023).

The results of this study together with the clinical and experimental ones carried out confirm that both freezing methods are useful for OCT. Therefore, it seems that the functionalism of ovarian tissue transplantation is fundamentally related to the local conditions of the transplantation site.

It is essential to note that, despite not achieving statistical significance, there was a slight alteration observed in the samples following the implementation of various cryopreservation methods. We acknowledge the limitations associated with the limited sample size in this study. Therefore, further investigations involving larger cohorts are necessary to validate and generalize these results. Additionally, more research is needed to explore the potential correlation with patients’ clinical characteristics and to ultimately confirm these findings. In conclusion, the results of this study illustrate that ovarian tissue freezing methods do not have a relevant impact on the viscoelasticity of the tissue and therefore do not seem to affect the functional characteristics of the ovarian stroma.

On the other hand, studies focused on the components of the ECM (matrisome) before and after transplantation would be necessary since its functionality is probably more related to these aspects than to the direct impact on the primordial follicles.
